# Evaluation of *Origanum vulgare* Essential Oil and Its Active Ingredients as Potential Drugs for the Treatment of Toxoplasmosis

**DOI:** 10.3389/fcimb.2021.793089

**Published:** 2021-11-22

**Authors:** Na Yao, Qiong Xu, Jia-Kang He, Ming Pan, Zhao-Feng Hou, Dan-Dan Liu, Jian-Ping Tao, Si-Yang Huang

**Affiliations:** ^1^ Institute of Comparative Medicine, College of Veterinary Medicine, Yangzhou University, Yangzhou, China; ^2^ Jiangsu Co-innovation Center for Prevention and Control of Important Animal Infectious Diseases and Zoonosis, Yangzhou, China; ^3^ Jiangsu Key Laboratory of Zoonosis, Yangzhou, China; ^4^ College of Animal Science and Technology, Guangxi University, Nanning, China; ^5^ Joint International Research Laboratory of Agriculture and Agri-Product Safety, the Ministry of Education of China, Yangzhou University, Yangzhou, China

**Keywords:** *Toxoplasma gondii*, natural medicine, *Origanum vulgare* essential oil, carvacrol, *in vitro*

## Abstract

*Toxoplasma gondii* is a serious hazard to public health and animal husbandry. Due to the current dilemma of treatment of toxoplasmosis, it is urgent to find new anti-*T. gondii* drugs to treat toxoplasmosis. In this study, the anti-*T. gondii* activity of Origanum vulgare essential oil (*Ov* EO) was firstly studied, and then, carvanol (Ca), the main ingredient of *Ov* EO was evaluated using the MTT assay on human foreskin fibroblast (HFF) cells *in vitro*. The cytotoxicity was evaluated using the MTT assay on HFF cells. The CC_50_ of *Ov* EO and Ca was 134.9 and 43.93 μg/ml, respectively. Both of them exhibited anti-parasitic activity, and inhibited the growth of *T. gondii* in a dose-dependent manner. For the inhibition effect, Ca was better than *Ov* EO at the same concentration, the IC_50_ of *Ov* EO and Ca was 16.08 and 7.688 μg/ml, respectively. In addition, treatment with Ca, was found to change the morphology of *T. gondii* tachyzoites and made their shapes curl up. These results showed that Ca was able to inhibit the proliferation of *T. gondii* by reducing invasion, which may be due to its detrimental effect on the mobility of tachyzoites. Our results indicated that Ca could be a potential new and effective drug for treating toxoplasmosis.

## Introduction

The opportunistic pathogen *Toxoplasma gondii* is a serious hazard to public health and animal husbandry (Chemoh et al., 2013). One-third of the people in the world have been infected by *T. gondii* where tachyzoites, cysts and oocysts are three infectious stages. Human intake of raw meat or water containing *T. gondii* cysts or oocysts can be infectious. In a few cases, direct contact with *T. gondii* tachyzoites between the mucous membrane and the damaged skin can also cause an infection. Cats are intermediate hosts of *T. gondii* and can rule out infectious oocysts. Accidental contact between humans and cat feces is a risk of infection. For most individuals with competent immunity, infection is asymptomatic and the *T. gondii* eventually lies dormant as a tissue cyst. For some people primary infection can cause ocular disease, and in pregnant women, it can lead to abortion, stillbirth or brain damage in a congenitally infected fetus. Recurrence of chronic infection is a frequent cause of toxoplasmic encephalitis (TE) in an immunosuppressive patient such as advanced HIV infection, neoplastic disease, or in those receiving immunosuppressive therapies (e.g., rituximab).

As *T. gondii* has a wide range of hosts, apart from humans, it also infects many animals, like cattle, sheep, pig and other domestic animals. This causes the economic loss of animal husbandry and the hidden danger of public food hygiene and safety.

The drug treatment of toxoplasmosis can be traced back to the use of sulfonamides in the 1940s. In the 1950s, sulfadiazine combined with pyrimethamine successfully treated toxoplasmosis in mice. It is still the golden treatment for toxoplasmosis today (Wei et al., 2015). However, the side effects and the emergence of drug resistance have undermined the perfection of the treatment regimen (Schmidt et al., 2006). In the case of pregnant women infected with *T. gondii*, spiramycin is a good drug for the treatment of toxoplasmosis because of its low toxicity and it cannot penetrate the placental barrier; however it has no effect on the infected fetus (Desmonts and Couvreur, 1974). Other drugs such as Trimethoprim-sulfamethoxazole, Clindamycin, and Atovaquone also have their own disadvantages (Dunay et al., 2018). Therefore, it is urgent to find new anti-*T. gondii* drugs with high efficiency and low toxicity to treat toxoplasmosis.

Natural products are one of the important sources of drug development (Petrovska, 2012). In the field of cancer treatment alone, from the 1940s to now, 48.6% of the 175 small molecules are natural products or obtained directly from there (Newman and Cragg, 2012). Plants as one of the natural products usually grow outdoors, so they have to resist the infection of disease and the pressure of harsh environment in the process of growing (Weng et al., 2012). In this process of defense, the molecules they produce give plants smell, color and even toxicity (Lietava, 1992). Essential oils are a mixture of these molecules, which are a potential drug reservoir.

*Origanum vulgare* that is native to the Mediterranean coast, North Africa and West Asia is a perennial herb of the genus Oregon of the *Lamiaceae* family (Elshafie et al., 2017). The *O. vulgare* essential oil (*Ov* EO) has been proven to have certain biological activity (Argyri et al., 2021). At a concentration of 60 μg/ml, *Ov* EO can inhibit the invasion rate of *Cryptosporidium parvum* into Human colon adenocarcinoma (HCT-8) cells by 60% (Gaur et al., 2018). *Ov* EO can also inhibit the growth of *Aeromonas hydrophila*, *Brevibacterium linens*, *Clostridium sporogenes*, *Leuconostoc cremoris*, and *Pseudomonas aeruginosa* (Dorman and Deans, 2000). The crude *Ov* EO can decrease the activity of liver cancer cells (HepG2 cell) in a dose-dependent manner, and the IC50 value was 236 μg/μl (Elshafie et al., 2017). In addition, *Ov* EO also shows excellent anti-inflammatory and antioxidant activities, and also has potential functions in controlling cardiovascular diseases and metabolic syndrome (Leyva-López et al., 2017).

In this study, the anti-*T. gondii* activity of *Ov* EO was firstly studied, and then the inhibited activity of carvanol (Ca), the main ingredient of *Ov* EO, was selected to evaluate *in vitro*.

## Materials and Methods

### Cell Culture and Parasites

*T. gondii* tachyzoites of the GFP-RH strain, expressing green fluorescence protein were proliferated in human foreskin fibroblast (HFF) cells, cultured in Dulbecco’s modified Eagle’s medium (DMEM), supplemented with 10% heat-inactivated fetal bovine serum (FBS) at 37°C, in an atmosphere containing 5% CO_2_. To isolate the tachyzoites, heavily infected cells were scraped and the parasites were released by passing the cells through a 27-gauge needle, three to five times. Cell debris was removed by passing the mixture through a 3-µm pore membrane filter (Whatman, ThermoFisher, Waltham, MA, USA). Tachyzoites were quantified using a hemocytometer before proceeding to further experiments.

### Essential Oil and Chemical Components

The *Ov* EO and Ca used in this experiment was provided by Guangxi University, EO was extracted by steam distillation and dissolved in dimethyl sulfoxide (DMSO) in a 1:1 ratio. Ca was dissolved into a suitable mother liquor with DMSO. The species number of *O. vulgare* used in this study is GXCM 2019023. The solutions were then diluted with DMEM, such that the final concentration of DMSO in the samples used in the experiment was lower than 1.56% v/v.

### Cytotoxicity Assay

The cytotoxicity of *Ov* EO and Ca was evaluated in an HFF cell line with a CellTiter 96^®^ AQueous One Solution Cell Proliferation Assay (Promega Corp., Madison, WI, USA), according to the manufacturer’s instructions. HFF cells (1 × 10^5^ cells/well) were seeded in 96-well plates and cultured at 37°C, in an atmosphere containing 5% CO_2_, for 24 h. The cells were treated with varying concentrations of *Ov* EO (70, 35, 17, 9, and 4 μg/ml), Ca (70, 35, 17, 9, and 4μg/ml) or sulfamethoxazole (SMZ), and a 1.56% solution of DMSO in DMEM was used as the vehicle control. After incubating for 24 h, the HFF cells viability were measured by the MTT (3-[4,5-methylthiazol-2-yl]-2,5-diphenyltetrazolium bromide) colorimetric method according to Costa et al. (2018). Approximately 20 μl of MTT solution (5 mg/ml) was added to each well and allowed to incubate at 37°C with 5% CO_2_ for 3 h and then 200 μl of DMSO was added to dissolve the formazan crystals. Absorbance was measured at 490 nm using an iMark™ Microplate Absorbance Reader (BioRad, Hercules, CA, USA). and the 50% cytotoxic concentrations (CC_50_) were calculated using Graph Pad Prism 8.0. The cytotoxicity experiment was performed in triplicate, using three separate plates.

### Anti-*T. gondii* Activity of *Ov* EO and Ca Evaluated by a Plaque Assay

One hundred freshly released GFP-RH tachyzoites were added to HFF monolayers in 6-well plates, in DMEM with 2% FBS. They were incubated at 37°C, in an atmosphere containing 5% CO_2_, for 4 h. Then, the extracellular parasites were removed with medium, and fresh medium containing various concentrations of *Ov* EOs, Ca or 1.56% DMSO (vehicle control) were added to each well. Uninfected and untreated wells were used as blank controls. After 7 days, HFF cells were washed three times with PBS, fixed with methanol for 10 min, and stained with 0.1% crystal violet for 30 min. After washing three times with phosphate buffered saline (PBS) and drying naturally (Huang et al., 2021), the plaques formed by tachyzoites were examined by microscopy.

### Anti-*T. gondii* Activity of *Ov* EO Evaluated by an Intracellular Growth Assay

HFF cells were incubated in 24-well plates for 48 h, then the medium was replaced by DMEM with 2% FBS, 100 freshly released GFP-RH tachyzoites were added to each well, and incubated at 37°C in an atmosphere containing 5% CO_2_, for 4 h. The medium containing extracellular parasites was removed and fresh medium containing either *Ov* EO (70, 35, 17, 9, and 4 μg/ml) or Ca (17, 9, 4, and 2 μg/ml), 1.56% DMSO (vehicle control), or 10 μg/ml SMZ (positive control) was added to each well. After 32 h, the growth of GFP-RH was observed and photographed under a fluorescence microscope. Growth of GFP-RH was calculated using Image-Pro-Express.

### Effect of *Ov* EO and Ca on the Invasion of *T. gondii*

Invasion experiments were performed as described by Augusto et al. (2018). HFF cells was cultured in a 6-well plate, and 3 ml DMEM with 2% FBS was added to each well. Then, 10^4^ RH and 17 μg/ml *Ov* EO or *Ca* were added simultaneously to the wells, respectively, incubating for 20, 40, or 60 min. The supernatant was gently removed, cells were fixed with 2 ml methanol for 10 min, washed three times with PBS, blocked by 5% solution of BSA in PBS (BSA/PBS) for 1 h, and washed three times with PBS. This was then incubated with mouse anti-*Toxoplasma* SAG1 monoclonal antibodies (mAb), diluted (1:1,000) with a 1% BSA/PBS solution, at room temperature for 2 h. Then, goat anti-mouse IgG H&L(FITC) secondary antibodies, diluted (1:1,000) in 1% BSA/PBS, were added to 6-well plates and incubated at room temperature for 2 h. After washing thrice with PBS, 300 μl of 0.2% Triton X-100 was added, and the mixture was left for 30 min. Cells were then gently washed three times with PBS, and 300 μl of a 5% BSA/PBS solution was added dropwise for a second blocking. The antibodies were added as per the procedure described earlier, this time using goat anti-mouse IgG H&L (Alexa Fluor ^®^ 568) (ab175473) instead of the goat anti-mouse IgG H&L (FITC). Finally, 300 μl of 30% glycerol was added to each well. Five visual fields were randomly selected for observation under the 40× objective of the fluorescence microscope and the parasites in each field were counted. Three repetitions were performed to increase the accuracy of the experiment. The difference between the tachyzoites of the two colors is termed as the absolute invasion number of tachyzoites. The ratio of the invasion number to the total number of tachyzoites is termed as the invasion rate of tachyzoites.

### Assessment of Tachyzoite Ultrastructure Using Scanning Electron Microscopy

To determine differences in the ultrastructure of tachyzoites after treatment, the purified tachyzoites were treated with 17 μg/ml *Ov* EO or Ca, respectively. After being cultured at 37 °C for 4 h, they were washed gently with PBS twice, and fixed overnight with 2.5% glutaraldehyde at room temperature. Gradient dehydration was carried out with 30, 50, 70, 80, 90, 95 and 100% ethanol respectively, and the critical point drying was carried out after dehydration. The tachyzoites were coated with gold (20−30 nm) and observed by scanning electron microscopy.

### Statistical Analysis

All data were analyzed using GraphPad Prism 8.0. The anti-parasitic activity of the *Ov* EO and Ca was analyzed using an unpaired *t*-test, while the cell invasion data were processed using multiple *t*-tests, to compare the results of the test groups and those of the control group (*P <0.05, **P <0.01, ***P <0.001).

## Results

### Cytotoxicity of *Ov* EO and Ca

**Figure 1:**
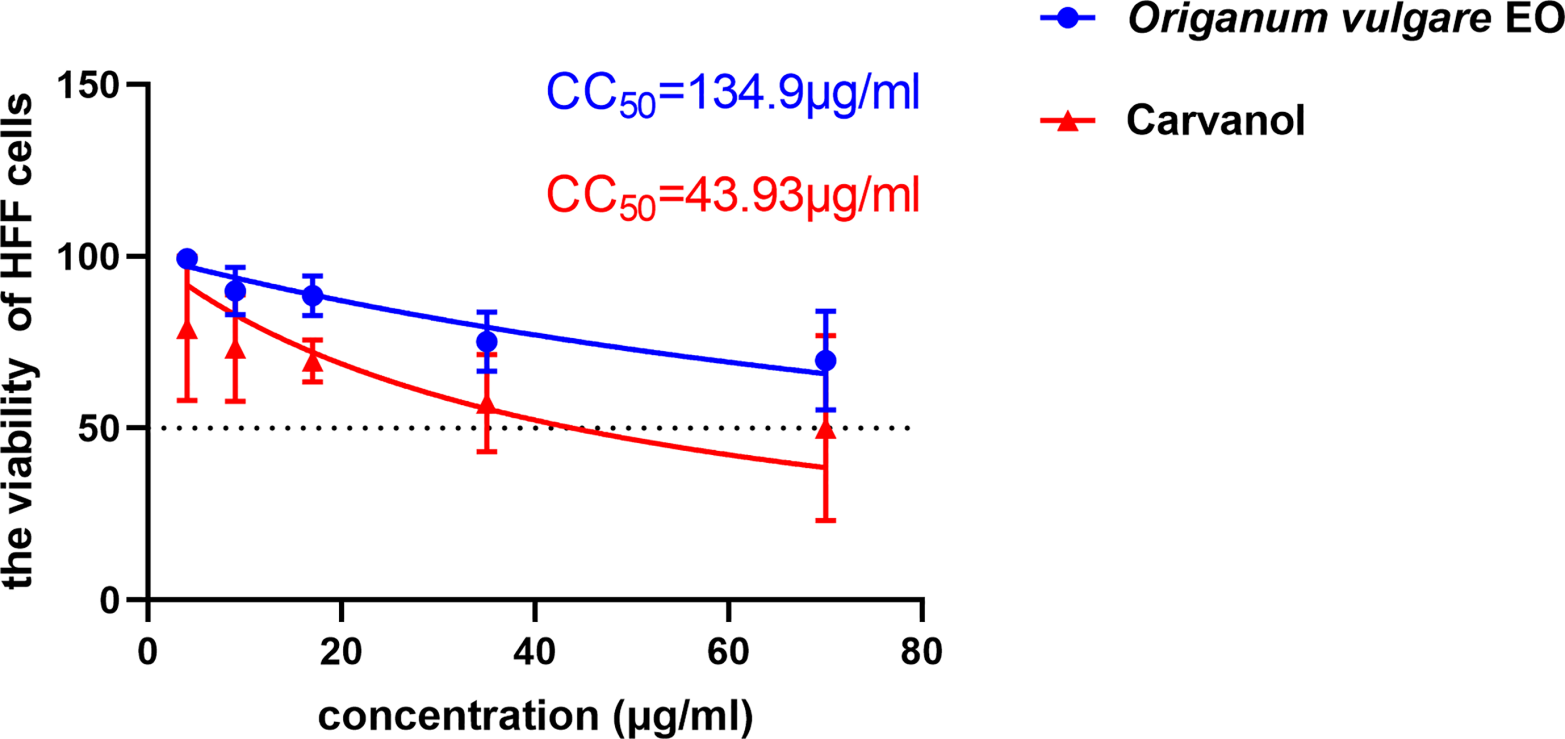
The 50% cytotoxic concentrations (CC_50_) of *Ov* EO and Ca. Cytotoxicity of *Ov* EO and Ca on HFF cells. Different concentrations of *Ov* EO *and* Ca treat HFF cells for 24 h and then cytotoxicity was evaluated using MTT Assay. All data are presented as with error bars and the experiments were performed in triplicate.

The cytotoxic potential of *Ov* EO on the host cell was confirmed before the antiparasitic activity study. According to MTT assay result, the concentration that induced 50% HFF cells mortality (CC_50_) of *Ov* EO was 134.9 μg/ml (**Figure 1**). After the antiparasitic activity of *Ov* EO was confirmed, the cytotoxic potential of Ca was carried out, and the result indicated that the CC_50_ of Ca was 43.93 μg/ml (**Figure 1**).

### Antiparasitic Activity of *Ov EO* and Ca *In Vitro*

**Figure 2:**
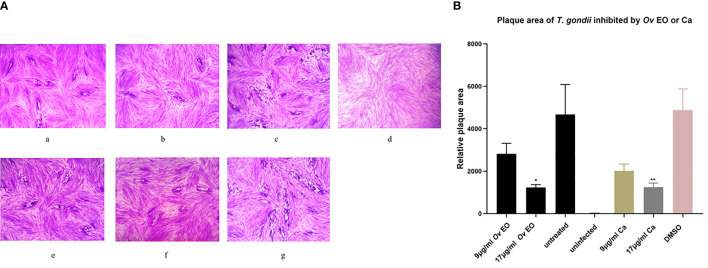
Plaque test for preliminary detection of anti-*T. gondii* activity of *Ov* EO and Ca. **(A)** Images of *T. gondii* plaque under different concentrations of *Ov* EO and Ca. **(B)** Statistical analysis of the images plaque. **(a)** HFF cells were infected by *T. gondii* and treated with 9 μg/ml *Ov* EO; **(b)** HFF cells were infected by *T. gondii* and treated with 17 μg/ml *Ov* EO; **(c)** HFF cells were infected by *T. gondii* and untreated; **(d)** HFF cells were not infected and treated; **(e)** HFF cells were infected by *T. gondii* and treated with 9 μg/ml Ca; **(f)** HFF cells were infected by *T. gondii* and treated with 17 μg/ml Ca; **(g)** HFF cells were infected by *T. gondii* and treated with 1.56% DMSO.

**Figure 3:**
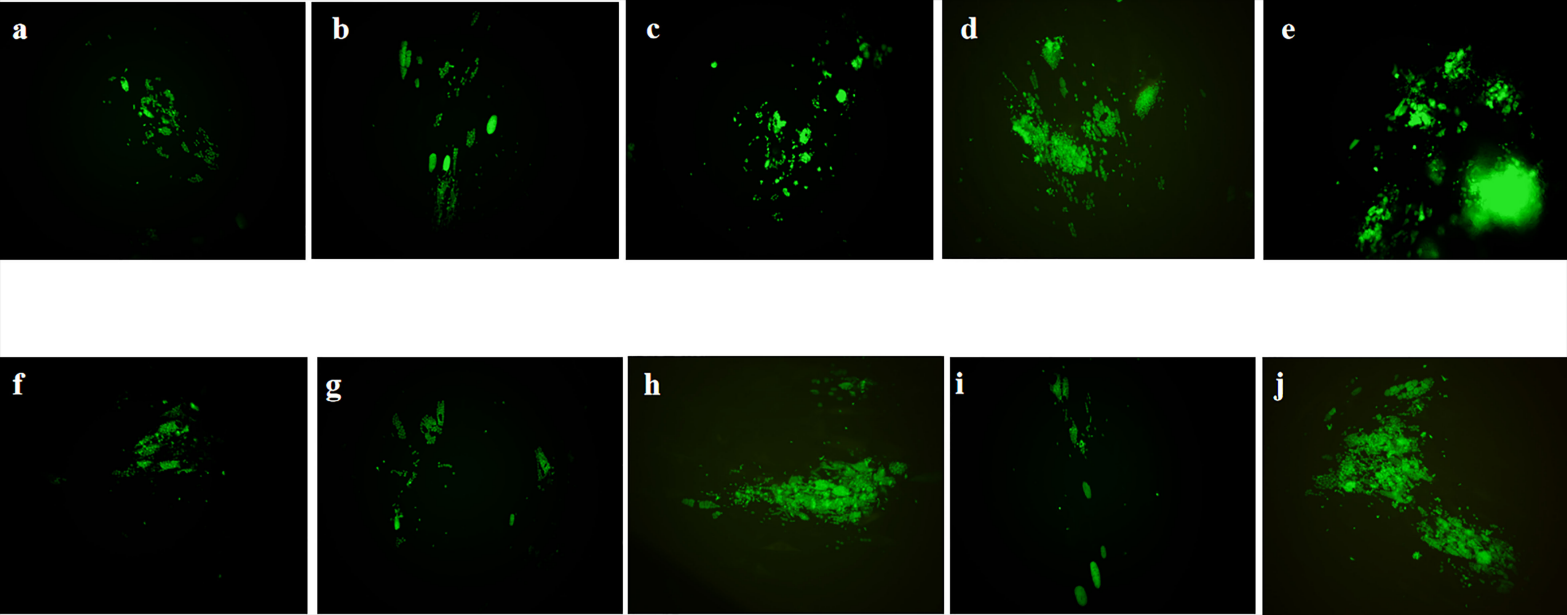
Anti-*T. gondii* activity of *Ov* EO and Ca evaluated by growth assay. Fluorescence area indicates the growth of *T. gondii* during different treatment. **(A–D)** Different concentrations of *Ov* EO, **(A)** 70 μg/ml, **(B)** 35 μg/ml, **(C)** 17 μg/ml, **(D)** 9 μg/ml, and **(E)** no treatment; **(F–H)** Different concentrations of Ca, **(F)** 17 μg/ml, **(G)** 9 μg/ml, **(H)** 4 μg/ml, **(I)** SMZ, and **(J)** DMSO.

**Figure 4:**
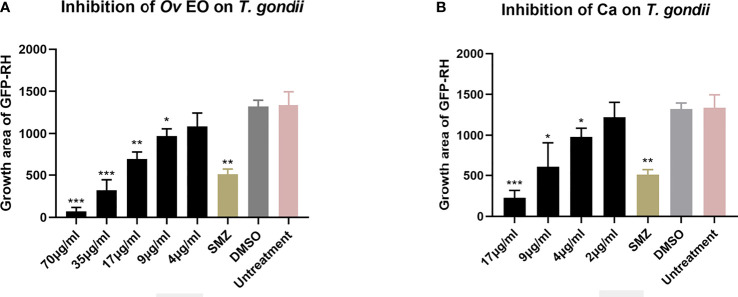
Statistical analysis of the inhibition effect of *Ov* EO and Ca in anti-*T. gondii*. Data analysis based on fluorescence area of GFP-RH. Each bar represents the mean ± SD of three wells per group. *P < 0.05, **P < 0.01, ***P < 0.001 compared with untreatment group. **(A)** Anti-*T. gondii* activity of *Ov* EO, and **(B)** Anti-*T. gondii* activity of Ca.

The anti-*T. gondii* activity of *Ov* EO was preliminarily evaluated by plaque test. As seen in **Figure 2A**, we found that the plaques visible were fewer in number and smaller in size after treatment with 9 or 17 μg/ml *Ov* EO (**Figures 2Aa, b**), as compared to those in the DMSO-treated and untreated groups. These results indicated that *Ov* EO was able to inhibit the growth of RH within safe concentrations. In order to find the effective ingredients in *Ov* EO that have the role of anti-toxoplasma, Ca was evaluated by plaque test. The results indicated that Ca was able to inhibit the growth of RH at 9 or 17 μg/ml (**Figures 2Ae, f**). The results indicated that the growth of *T. gondii* was inhibited by each of the concentrations of *Ov* EO and Ca tested (**Figure 2B**). To confirm and evaluate the effect of *Ov* EO concentration on anti-parasitic activity, five different concentrations were compared using an *in vitro* inhibition assay. The results indicated that the growth of *T. gondii* was inhibited by each of the concentrations of *Ov* EO tested (**Figure 3**), and the inhibition increased in a dose-dependent manner (**Figure 4A**), the IC_50_ of *Ov* EO was 16.08 μg/ml. We could find that the growth of *T. gondii* was significantly reduced after treatment with 70 and 35 μg/ml *Ov* EO (71 *vs*. 1,337; 320 *vs*. 1,337, *P <*0.001), as compared to the untreated and 1.56% DMSO-treated groups.

**Figure 5:**
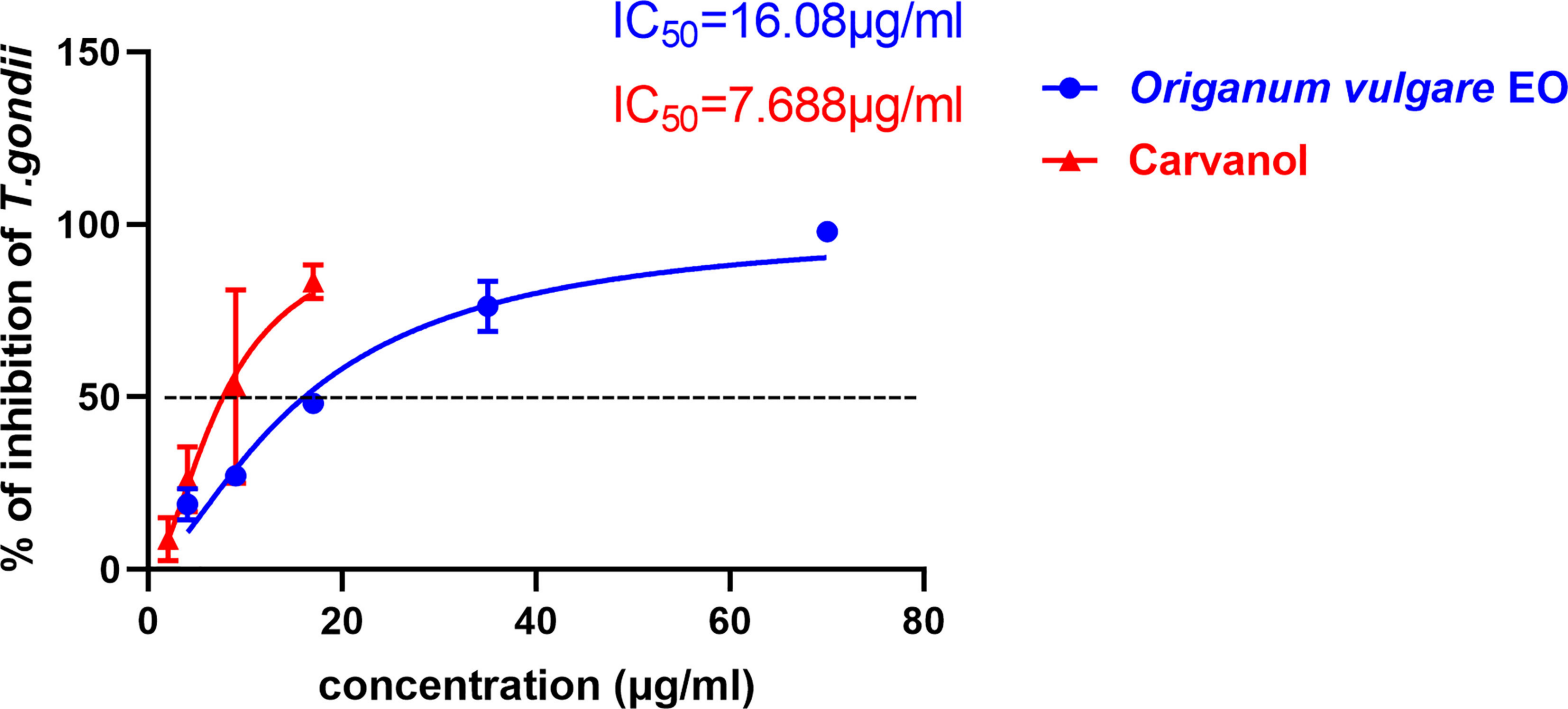
The 50% inhibition concentrations (CC_50_) of *Ov* EO and Ca. Inhibition of *Ov* EO and Ca to *T. gondii*. Different concentrations of *Ov* EO *and* Ca treated infected HFF cells for 32 h, the growth of GFP-RH was observed and photographed under a fluorescence microscope. Growth of GFP-RH was calculated using Image-Pro-Express. All data are presented as with error bars and the experiments were performed in triplicate.

To compare the effect between *Ov* EO and Ca on anti-parasitic activity, four different concentrations of Ca were compared using the same assay. The results indicated that the growth of *T. gondii* was inhibited by each of the concentrations of Ca tested (**Figure 3**), and the inhibition also increased in a dose-dependent manner (**Figure 4B**), the IC_50_ of Ca was 7.688 μg/ml. Comparing to the untreated group, the growth of *T. gondii* was also significantly reduced in low Ca concentrations treated groups (9 and 4 μg/ml) (606.8 vs 1,337; 980.7 *vs*. 1,337, P <0.05). Both *Ov* EO and Ca inhibited the growth of *T. gondii* in a dose-dependent manner, for the groups treated with 17 μg/ml *Ov* EO and 17 μg/ml Ca, the differences were also significant (692.9 *vs*. 1,337, *P <*0.01; 225.5vs. 1,337, *P <*0.001). For the inhibition effect, Ca was better than *Ov* EO at the same concentration. The inhibition of *T. gondii* was much more significant in the groups treated with 17 μg/ml Ca, than in those treated with SMZ (225.5 *vs*. 1,337, *P <*0.001; 490 *vs*. 1,337, *P <*0.01). There was no significant decrease in untreatment groups after treatment with 1.56% DMSO, which indicated that DMSO had no inhibitory effect on GFP-RH (1,322 vs 1,337). Due to the results of inhibition, we can calculate the IC 50 of *Ov* EO and Ca was 16.08 and 7.688 μg/ml, respectively (**Figure 5**). After statistical analysis, the selectivity index (SI) of *Ov* EO and Ca was 8.389 and 5.714, respectively (**Table 1**). From the comprehensive comparison of safety, the performance of *Ov* EO is better than that of Ca.

**Table 1:** Anti-*T. gondii* activity and cytotoxic effects of *Ov* EO or Ca.

	CC_50_ (μg/ml) (95% Confidence Intervals)HFF	IC_50_ (μg/ml) (95% Confidence Intervals)*T. gondii*	Selectivity Index (SI) (CC_50_/IC_50_)
*Ov* EO	134.9 (102.6–184.9)	16.08 (13.88–18.55)	8.389
Ca	43.93 (26.87–75.07)	7.688 (5.711–10.33)	5.714

### Effect of *Ov* EO and Ca on the Invasion of *T. gondii*

**Figure 6:**
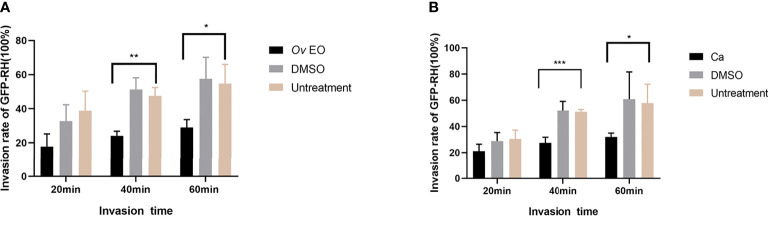
Effect of *Ov* EO on the invasion of *T. gondii.* Statistics of *T. gondii* invasion rate using two immunofluorescent dyes after treated with *Ov* EO **(A)** and Ca **(B)** for 20, 40, 60 min, respectively. *P < 0.05, **P < 0.01, ***P < 0.001 compared with untreated group.

As summarized in **Figure 6**, in the 17 μg/ml *Ov* EO treatment group, the *T. gondii* invasion rates at 20, 40, and 60 min post-infection were found to be 17.84, 24.10, and 28.96% respectively (**Figure 6A**). In the untreated group, invasion rates were found to be 38.85, 47.52, and 54.70% respectively at the three time points (**Figure 5A**). The invasion of *T. gondii* was significantly inhibited by *Ov* EO at 40 *min* (24.10% *vs*. 47.52%, *P <*0.01) and 60 min (28.96% *vs*. 54.70%, *P <*0.05). In the 17 μg/ml Ca treatment group, the *T. gondii* invasion rates were 21.09, 27.51, and 32.03% respectively at the three time points (**Figure 6B**), and the control group, invasion rates were found to be 30.52, 51.20, and 57.81% respectively (**Figure 6B**). Compared to the untreated group. Ca significantly reduced the invasion of *T. gondii*, especially after treatment for 40 and 60 min (27.51% *vs*. 51.20%, *P <*0.001; 32.03% *vs*. 57.81%, *P <*0.05, **Figure 6B**). The inhibitory effect was observed to increase as the treatment time increased. No change in the invasion rate of *T. gondii* was observed in any group treated with DMSO, across all experiments.

### Tachyzoite Ultrastructure Analysis

**Figure 7:**
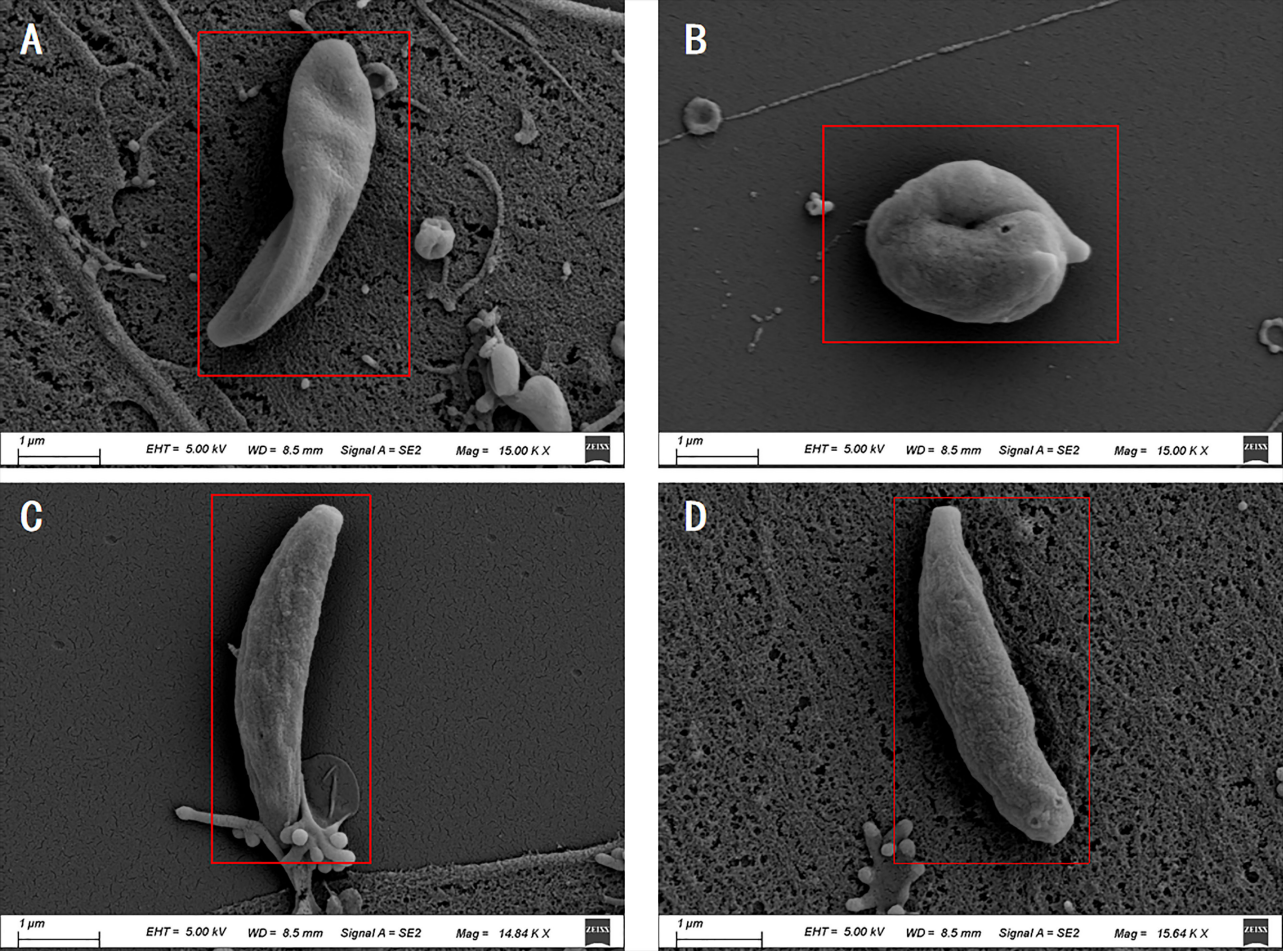
Scanning electron microscopy assay. The *T. gondii* were treated with 17 μg/ml *Ov* EO **(A)**, 17 μg/ml Ca **(B)**, DMSO **(C)** or untreated **(D)**. After treated by *Ov* EO, the tachyzoites became sunken compared with those untreated tachyzoites. Tachyzoites treated with Ca become curled up. Scale bars: 1 μm.

The Scanning electron microscopy (SEM) results showed that the tachyzoites curled into a head-to-tail shape after the Ca treatment, but the individual was still plump and not shriveled (**Figure 7B**). After treatment with *Ov* EO, the morphology of the tachyzoites changed significantly, the worms were distorted, and showed a certain shriveled state (**Figure 7A**). In comparison, the tachyzoites were normal full crescent-shaped in DMSO treated group (**Figure 7C**) and untreated group (**Figure 7D**).

## Discussion

*T. gondii* is an important zoonotic parasite, which can cause serious consequences after infected different hosts (Elsheikha, 2008; Maldonado and Read, 2017; El Bissati et al., 2018). In the face of the current dilemma of treatment and prevention, it is urgent to explore new drugs to inhibit *T. gondii* and control toxoplasmosis. At present, several reports indicated that plant essential oils had the inhibitory effect on *T. gondii*, in which, *Bunium persicum* (Boiss) EO (Tavakoli Kareshk et al., 2015) and *Thymus broussonetii Boiss* EO (Dahbi et al., 2010) played obvious roles in acute and chronic *T. gondii* infection in mice respectively. *Ov* EO is an important condiment, perfume, cosmetics, incense and preservative, with a long history of application (Diniz do Nascimento et al., 2020). The composition and content of *Ov* EO are affected by many factors, such as the place of production, cultivation conditions, extraction technology, etc. Research data shows that Ca is an important component in *Ov* EO, and its content mainly depends on the place of production, The carvacrol content in *Ov* EO produced in Argentina, Brazil, Greece and China is 26.7–81.92, 73.9, 63.03, and 30.73%, respectively (Leyva-López et al., 2017). This was the reason we chose the Ca study whether it plays an anti-*Toxoplasma* effect in *Ov* EO.

The cytotoxicity of *Ov* EO is related to the extraction method, and its toxicity to different cell lines is also different. The cytotoxicity of methanolic extracted *Ov* EO was more toxic, the CC_50_ of it was 382–374 μg/ml in MCF-7 cells. Chaouki reported that the CC_50_ of *Ov* EO was 5.5, 5.2, and 7.5 μg/ml in breast cancer cells (MCF-7), lung cancer cells (H-460) and central nervous system cancer cells (SF-268), respectively (Chaouki et al., 2010). In our experiment, the CC_50_ of *Ov* EO was 134.9 μg/mL in HFF. It has quite low cytotoxicity, so we can continue to carry out follow-up studies to evaluate its anti-*Toxoplasma* effect. The CC_50_ of Ca was 43.93 μg/ml, which is more toxic than *Ov* EO in HFF cells. Mostly, the active ingredients are more toxic than essential oil mixtures, and the effect will be better. From our results we found that the SI of *Ov* EO and Ca was not very high, which means that the cytotoxic of them are quite high, so further study should be carry on to reduce their cytotoxicity. Gaur et al. (2018) also found that Ca was more toxic than *Ov* EO in HCT-8 cells. In our experiment, Ca showed better anti-*T. gondii* activity than *Ov* EO at same concentration. As a kind of phenol, Ca has a significant repellent effect on many kinds of insects, such as *Aedes albopictus*, *Culex quinquefasciatus*, *Ixodes scapularis*, *Rhipicephalus appendiculatus* and so on (Lima et al., 2019). Moreover, Ca also shows anti-nematode effect on different nematodes such as *Ascaris suum* (Trailović et al., 2015). The main reason is that Ca can inhibit the contraction of muscle cells induced by acetylcholine, thereby inhibiting the muscle contraction of the parasite and affecting its motility (Marjanović et al., 2020). The above speculation is that the mechanism of carvacrol against *T. gondii* is related to the restriction of the motility of the parasite. However, its effect on the stability of calcium ions is also worthy of attention. Carvacrol has a certain regulatory effect on the stability of intracellular calcium ions. Studies have shown that carvacrol can adjust the TRP channels of transient calcium permeation receptor potentials, such as TRPV3, thereby increasing the concentration of calcium ions in the cytoplasm of primary mouse corneal epithelial cells and cultured human corneal epithelial cells (HCE-T cells) (Yamada et al., 2010). As we all know, there is a very important Ser/Thr protein kinase family in *T. gondii*, the CDPK family, whose activity is directly regulated by calcium ions. One family member, CDPK1, is closely related to the adhesion and invasion of *T. gondii*. Therefore, the reason why carvacrol can inhibit *T. gondii* may be achieved by inhibiting the activity of its invasion-related proteins. In our results, Ca did significantly inhibit the invasion rate of tachyzoites on HFF cells.

According to the results of SEM, the tachyzoites curled into a head-to-tail shape after the Ca treatment. Obviously, this obvious morphological change caused damage to the mobility of tachyzoites, which in turn affected its ability to invade. Studies have shown that carvacrol may induce apoptosis by reducing mitochondrial potential, releasing cytochrome C, activating caspase and carving PARP, thereby inhibiting human metastatic breast cancer cell line (MDA-MB 231) or human non-small cell lung cancer cell line Proliferation (A549) (Suntres et al., 2015). Therefore, the specific mechanism of Ca inhibiting *T. gondii* is not yet fully understood, and further research is needed. After *Ov EO* treatment, the tachyzoites showed severe dehydration and dryness. *Ov EO* is a mixture of different components, including terpenes, aldehydes, alcohols, esters, phenolic, ethers, and ketones and so on (Swamy et al., 2016). Among them, phenol can dehydrate the cells, resulting in the desiccation phenomenon (Samie et al., 2019). In addition, some hydrophobic compounds can pass through biological barriers and biological membranes, which may also have anti-*T. gondii* effects (Costa et al., 2018).

## Conclusion

In this study, we found that *Ov* EO and Ca had obvious anti-*Toxoplasma* effect, which is likely to be achieved by changing the shape of tachyzoites, thereby limiting its movement ability, and then affecting its invasion ability. At the same time, Ca may have other biological functions, which can inhibit the proliferation of *T. gondii*. However, the target molecular and mechanism of action of *Ov* EO on *T. gondii* are still unclear and warrant further studies.

## Data Availability Statement

The raw data supporting the conclusions of this article will be made available by the authors, without undue reservation.

## Author Contributions

S-YH and NY conceived and designed the study. NY, QX, J-KH, MP, and Z-FH performed the laboratory analyses. D-DL and JPT analyzed the data. All authors critically appraised and interpreted the results. NY drafted the first version of the manuscript. All authors provided feedback on the manuscript, and read and approved the final version.

## Funding

The sample collection and some experiments were supported by the Outstanding Youth Foundation of Jiangsu Province of China (BK20190046), the China Postdoctoral Science Foundation (2020M671615), the Science and Technology Major Project of Zhejiang Province, China. (No. 2012C12009-2), and the Priority Academic Program Development of Jiangsu Higher Education Institutions (Veterinary Medicine).

## Conflict of Interest

The authors declare that the research was conducted in the absence of any commercial or financial relationships that could be construed as a potential conflict of interest.

## Publisher’s Note

All claims expressed in this article are solely those of the authors and do not necessarily represent those of their affiliated organizations, or those of the publisher, the editors and the reviewers. Any product that may be evaluated in this article, or claim that may be made by its manufacturer, is not guaranteed or endorsed by the publisher.
